# Stabilization of the Single-Chain Fragment Variable by an Interdomain Disulfide Bond and Its Effect on Antibody Affinity

**DOI:** 10.3390/ijms12010001

**Published:** 2010-12-23

**Authors:** Jian-Xin Zhao, Lian Yang, Zhen-Nan Gu, Hai-Qin Chen, Feng-Wei Tian, Yong-Quan Chen, Hao Zhang, Wei Chen

**Affiliations:** 1 School of Food Science and Technology, Jiangnan University, Wuxi, Jiangsu, 214122, China; E-Mails: jxzhao@jiangnan.edu.cn (J.-X.Z.); yanglian27@yahoo.com.cn (L.Y.); haiqinchen@jiangnan.edu.cn (H.-Q.C.); fwtian@jiangnan.edu.cn (F.-W.T.); 2 State Key Laboratory of Food Science and Technology, Jiangnan University, Wuxi, Jiangsu, 214122, China; E-Mail: zhanghao@jiangnan.edu.cn (H.Z.); 3 Department of Cancer Biology, School of Medicine, Wake Forest University, Winston Salem, NC 27109, USA; E-Mails: zgu@wfubmc.edu (Z.-N.G.); yqchen@wfubmc.edu (Y.-Q.C.)

**Keywords:** aflatoxin, affinity, disulfide bond, single-chain fragment variable, stability

## Abstract

The interdomain instability of single-chain fragment variable (scFv) might result in intermolecular aggregation and loss of function. In the present study, we stabilized H4—an anti-aflatoxin B_1_ (AFB_1_) scFv—with an interdomain disulfide bond and studied the effect of the disulfide bond on antibody affinity. With homology modeling and molecular docking, we designed a scFv containing an interdomain disulfide bond between the residues H44 and L100. The stability of scFv (H4) increased from a GdnHCl_50_ of 2.4 M to 4.2 M after addition of the H44-L100 disulfide bond. Size exclusion chromatography revealed that the scFv (H44-L100) mutant existed primarily as a monomer, and no aggregates were detected. An affinity assay indicated that scFv (H4) and the scFv (H44-L100) mutant had similar IC_50_ values and affinity to AFB_1_. Our results indicate that interdomain disulfide bonds could stabilize scFv without affecting affinity.

## 1. Introduction

Recombinant antibodies are used in research, diagnostic, and therapeutic applications. The relatively easy production of the single-chain fragment variable (scFv) antibody in bacteria and affinity maturation explain the increasing importance of scFv in many applications. Unfortunately, many scFvs are not stable and tend to unfold or aggregate in practice [1]. Thus, improving the stability of scFv is a major challenge for protein engineers.

Insufficient interface stability between the heavy and light chains of scFv fragments has often been suggested to be the main cause of irreversible scFv inactivation. Fv fragments have been reported to dissociate into heavy-chain variable domains (VH) and light-chain variable domains (VL) with *K*_D_ values ranging from 10^−9^ to 10^−6^ M [2]. An antibody with improved stability could be generated by increasing the favorable interactions at the interface between the VH and VL domains; however, such antibodies are difficult to create [3]. In addition to the linker between the VH and VL domains, an interdomain disulfide bond was designed to stabilize the scFv [4]. Few reports described the effect of the interdomain disulfide bond on antibody affinity [5].

Using molecular modeling, we designed an interdomain disulfide bond in an anti-aflatoxin B_1_ (AFB_1_) scFv to improve its stability. We studied the affinity and stability of a scFv (H4) [6] against aflatoxin B_1_ (AFB_1_) and an interdomain disulfide bridge containing a mutation of H44-L100, which were expressed in functional form in *Escherichia coli* (*E. coli*).

## 2. Results

### 2.1. Modeling of scFv and Design of the Interdomain Disulfide Bond

**Figure 1:**
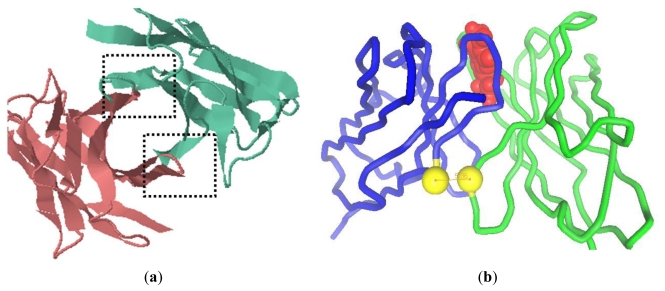
The computer modeling of scFv (H4) structure and design of interdomain disulfide bond between H44 and L100. (**a**) The vertical view of model of scFv (H4). The dashed boxes indicate the interface between VH and VL; (**b**) a pipe diagram of the model of H44-L100 (red: AFB_1_, blue: VL, green: VH and yellow: sulfide atom).

On the basis of alignment results, the VL framework structure of scFv (H4) was constructed with the consensus of an anti-human vascular endothelial growth factor Fab (PBD code: 1za3) with a resolution of 3.35 Å [6]. The VH (H4) domain was constructed by referring to the consensus structure of the anti-sodium citrate symporter CitS Fab (PBD code: 2v7n) with a resolution of 1.92 Å [7]. Both the H4 domains share more than 90% sequence identity with the corresponding domains of the antibodies. We took the previously resolved structures into account [7–10] and predicted that scFv (H4) has a tubiform structure. The complementary-determining regions (CDRs) formed loops at the tip of each domain and were supported by the structurally conserved framework region [11,12]. Two potential locations for the interdomain bond were identified on the basis of domains proximity, evidenced in the vertical view of the model (indicated by the dashed box in Figure 1a). The shortest distance was measured between framework H2 and framework L4, but also framework L2 and framework H4 were sufficiently close for a putative disulfide bridge. Some amino acid residue pairs were designed to introduce the interdomain disulfide bond, and the distance between the Cα atoms of these pairs was evaluated (Table 1). Previous research showed that the Cα-Cα distance of the disulfide bond in cysteine residues in known proteins ranges from 4.2 Å to 6.6 Å [8,13]. H44-L100, H46-L98, and H103-L43 were the possible sites with an optimal Cα-Cα distance [14]. However, disulfide bonds of H46-L98 and H103-L43 were very close to the CDR L3 (from L89 to L97) and CDR H3 (from H95 to H102) [15], respectively. H44-L100 was selected since it possessed the shortest distance between the two sulfide atoms and was sufficiently distant from the CDRs to prevent possible interference with the antibody binding capacity. In scFv (H4), the substitution of Gly with Cys at the site of H44 introduced a slight structure stress, neutralized by the Gly at H42. Similarly, the substitution of Gln with Cys at L100 would not significantly affect the domain folding due to the neighboring Gly residues at L99 and L101. Hence the introduction of the two cysteines produced a scFv (H44-L100) mutant with a general conformation similar to the cognate scFv (H4).

According to molecular docking, the interdomain disulfide bond of H44-L100 is between structurally conserved framework positions, which are distant from the antigen binding sites (Figure 1b). According to the software, the affinity of scFv (H4) and the scFv (H44-L100) mutant to AFB_1_ was 1.6 μM and 1.4 μM, respectively.

### 2.2. Expression of Antibody Fragments by E. coli

The expression of scFv in bacteria often encounters the formation of inclusion bodies, which can occur due to the reducing environment in the cytoplasm, which does not allow protein folding stabilization through disulfide bonds [16]. In our experiment, scFv (H4) and its derivates were secreted into the periplasm in a soluble form. Functional H4 was expressed at a concentration of 36 mg/L of the culture medium, while the amount of functional scFv (H44-L100) mutant was 8.85 mg/L of the culture medium. No free sulfhydryl group was found, indicating that the all the six sulfhydryl groups existed in disulfide bond form. The functionality of scFv (H44-L00) suggested correct formation of the native intradomain disulfide bonds in VH and VL, which was necessary for the functional conformation of scFv. Hence, we deduced that the sulfhydryl groups of the mutation cys were involved the interdomain disulfide bond.

### 2.3. Aggregation Behavior

**Figure 2:**
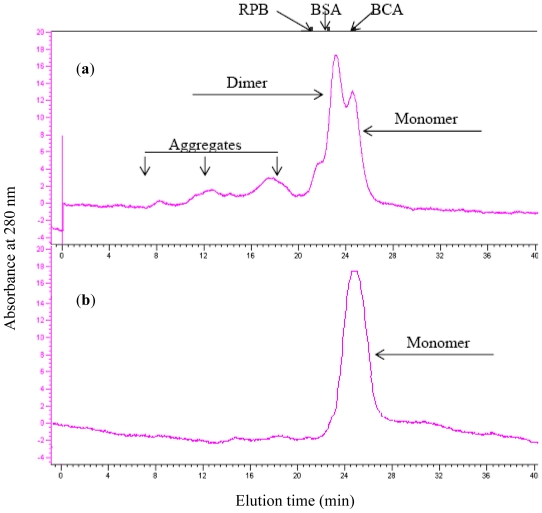
Aggregation of scFv (H4) (**a**, 10 μM) and scFv (H44-L100) mutant (**b**, 10 μM) after incubation at 37 °C for 20 h. The amount of monomeric scFv fragment was quantified by integrating the peaks representing monomer, which was separated by a Superdex-75 analytical gel filtration column. Elution volumes of molecular mass marker proteins are indicated: bovine carbonic anhydrase (BCA, 31 KDa), bovine serum albumin (BSA, 66 KDa), and rabbit phosphorglase B (RPB, 97 KDa).

Aggregation of scFv might result in the loss of functionality and interference in the study of the corresponding monomer [4]. Aggregation was concentration-dependent, and oligomerization of scFv (H4) and the scFv (H44-L100) mutant at various concentrations was assessed by size exclusion chromatography. For scFv (H4), the monomer fraction accounted for approximately 30% of the total protein at 10 μM (Figure 2a). H44-L100 existed primarily as monomeric proteins at 10 μM and aggregated forms were not detectable (Figure 2b). As shown by the results of the unfolding study (Figure 2), the aggregation of antibody fragments was negligible when the concentration of scFv was less than 1 mM.

### 2.4. Guanidinium Chloride (GdnHCl)-Induced Unfolding Profiles

**Figure 3:**
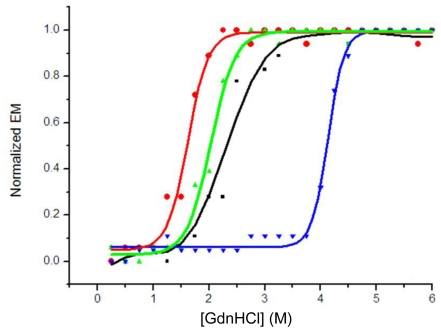
GdnHCl-induced unfolding of scFv (H4) (▪), VL (H4) (●), VH (H4) (▴) and scFv (H44-L100) (▾). Unfolding was monitored by fluorescence emission maximum (λ_max_, excitation at 280 nm) and is presented as normalized λ_max_. The λ_max_ of folded protein was 337 nm and λ_max_ of completely unfolded protein was 351 nm. Normalized curves are shown to facilitate the comparison of the unfolding transitions. All the presented data are the average of duplicates.

The wavelength at the maximum emission of the fluorescence spectrum (λ_max_) was used in the unfolding study, because the emission maximum depends solely on the environment of the tryptophan residues detected and is not affected by the protein concentration during unfolding [3,17,18]. The half-denaturation concentration of GdnHCl (GdnHCl_50_) was used to describe the stability of the protein in the unfolding experiment. A comparison of the denaturizing profiles of isolated domains indicated that VH (H4) with a GdnHCl_50_ of 1.9 M was more stable than VL (H4) with a GdnHCl_50_ of 1.5 M; their overlapped unfolding transition resulted in a broader unfolding transition of scFv (H4). A GdnHCl_50_ of 2.4 M for scFv (H4) suggested co-operative unfolding of two domains in scFv (Figure 3), which was higher than those of the isolated domains. The stability of scFv (H4) was increased by the introduction of an interdomain disulfide bond, and the scFv (H44-L100) mutant had a GdnHCl_50_ of 4.2 M.

### 2.5. Effect of Disulfide Bond on Binding Activity

**Figure 4:**
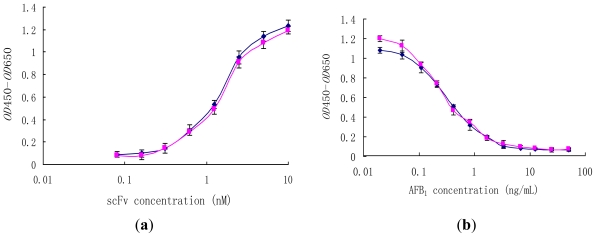
Binding behaviors of scFv (H4) (♦) and the scFv (H44-L100) (■) mutant in non-competitive ELISA (**a**) and competitive ELISA (**b**). (**a**) Wells were coated with 25 μL, 50 μM AFB_1_-BSA; (**b**) 2.5 μM scFv was preincubated with free AFB_1_ (0.02–50 ng/mL) at 37 °C for 1 h.

Generally, previous studies on the effect of interdomain disulfide bonds focused on stability, while their effect on antibody affinity was not well documented. The reactivity of scFv (H4) and the scFv (H44-L100) mutant was determined by direct and competition ELISA (Figure 4). In non-competitive ELISA studies, scFv (H4) and scFv (H44-L100) were able to bind to AFB_1_-BSA (50 μM) coated on microtiter plates in a concentration-dependant manner (Figure 4a) and they showed similar binding behaviors.

Comparable ELISA results were obtained with both scFv (H4) and the scFv (H44-L100) mutant. Pre-incubation of scFvs (10 μM) with free AFB_1_ resulted in the inhibition of scFvs binding to the microtiter plates coated with AFB_1_-BSA, and the binding profiles of scFv (H4) and scFv (H44-L100) mutant to free AFB_1_ were statistically identical (Figure 4b). The IC_50_ values of both scFv were approximately 0.4 ng/mL.

## 3. Experimental Section

### 3.1. Bacterial Strains, Vectors, Enzymes, and Chemicals

The recombinant antibody clone scFv (H4) consisting of the anti-AFB_1_ scFv was selected from the Tomlinson J Library (Geneservice, Cambridge, U.K.) [6]. *E. coli* BL21 (DE3) and pET22b vector were from Novagen (Beijing, China). All the enzymes used in this study were obtained from New England Biolabs (Beijing, China). AFB_1_ and aflatoxin B_1_-bovine serum albumin (AFB_1_-BSA) conjugates were purchased from Sigma (Shanghai, China). Other chemicals were supplied by Sangon (Shanghai, China).

### 3.2. Homology Modeling and Molecular Docking

The model of H4 was performed using WAM^®^ algorithm [19,20]. The dead-end eliminations method was used for side-chain building, and root mean square deviation was used to screen the final model. Molecular docking was processed with a PatchDock^®^ server and refined with a FireDock sever. The docking solution was evaluated using the Logplot software [21]. The docking solution was visualized using the program 3DMol^®^ viewer (Informax Inc.); distance measurements were carried out with the same software package.

### 3.3. Cloning and Construction of scFv

The H4 gene was amplified by polymerase chain reaction (PCR) with the primers LMB and pHEN (Table 2), and the PCR product was double digested with the restriction enzymes *Nco*I and *Not*I. The enzymatically digested scFv gene fragment was linked with pET22b (+) and then transformed into *E. coli* BL21 (DE3). Restriction enzyme digestion and DNA sequencing (Sangon, Shanghai) confirmed colonies bearing the pET22b/H4 construct.

The scFv (H44-L100) mutant was constructed with the introduction of cysteine residues to H44 and L100 by PCR with clone H4 as the template. PCR A was run with primers H44For and L100Back, while PCR B was run with LMB and H44Back. The 2 target segments were linked by PCR without primers for 5 cycles, and then amplified in another 35 cycles after addition of the primers of LMB and L100Back. The resulting segment was digested with *Nco*I and *Not*I and cloned to pET22b (+), as described above.

### 3.4. Expression and Purification of scFv

The expression and purification of scFv in *E. coli* BL21 (DE3) was performed according to the methods described in the pET^®^ system manual. Briefly, a single clone of BL21 (DE3)/pET22b/H4 was selected and grown to mid-logarithmic phase (OD600 = 0.8), after which IPTG was added to a final concentration of 1 mM. The cells were allowed to grow at 20 °C for another 20 h. The cells were harvested by centrifuging at 3300 × g for 15 min. The resulting pellet was resuspended in 10 mL of phosphate-buffered saline (PBS) and ultrasonicated in an ice bath. The soluble scFv was purified using Ni-chelating affinity chromatography (GE Healthcare, Shanghai).

### 3.5. Determination of Free Sulfhydryl Groups

The sulfhydryl content in scFv (H4) and the scFv (H44-L100) mutant was determined by mixing 70 μL of scFv (10 mg/mL) with 1 mL of freshly prepared 2-nitro-5-thiosulfobenzoate (NSTB) test solution. The absorbance at 412 nm was determined using the NSTB solution as a reference [22].

### 3.6. Analytical Gel Filtration

Gel filtration of scFv (H4) and the scFv (H44-L100) mutant were performed with the AKTA^®^ system using a column of Superdex^®^ 75 (Tricorn, Amersham Bioscience). All measurements were carried out in PBS containing 0.005% Tween 20. One milliliter of scFv fragments (10 μmol/L) was injected in the column. All the purified scFv fragments were analyzed after incubation at 37 °C for 20 h. The incubated samples were centrifuged at 14,000 × g for 5 min before loading to remove the insoluble aggregates.

### 3.7. Fluorescence Measurement

All fluorescence measurements were performed with a fluorescence spectrophotometer (Hitachi 650-60) at 25 °C. The protein was excited at 280 nm and the emission spectrum was recorded from 300 nm to 380 nm. For the equilibrium denaturation measurements, the protein sample (0.5 μM) was incubated at 16 °C for 20 h in PBS containing different amounts of GdnHCl.

### 3.8. Enzyme-Linked Immunosorbent Assay

Maxisorp^®^ plates were coated with 25 μL of AFB_1_-BSA (5 μg/mL) at 37 °C for 2 h or with 25 μL of BSA (5 μg/mL) as negative control [18]. The coated wells were washed with PBS twice, blocked with 2% BSA at 37 °C for 2 h, and washed again with PBS. Fifty milliliters of the antibodies amplified from each round of selection were mixed with 100 μL of 2% BSA and incubated at 37 °C for 1 h, after 4 washes with PBS containing 0.1% Tween 20 (PBST). Protein A/HRP antibody in 2% BSA (1:5000 dilution) was added and incubated at 37 °C for 1 h. The plate was washed again with PBST 4 times. The signal was visualized with 100 μL of 3,3',5,5'-tetramethylbenzidine (TMB, 100 μg/mL) in acetate buffer (pH 5.5). The reaction was stopped by adding 50 μL of 1 M H_2_SO_4_, and the optical densities at 450 nm (OD450) and 650 nm (OD650) were recorded with a MK3^®^ microplate reader (Thermo, Shanghai, China). Individual clones from the titer plates of the third selection round were randomly chosen, amplified in phage form, and used in monoclonal phage enzyme-linked immunosorbent assay (ELISA).

In the competitive ELISA, Maxisorp^®^ plates were coated with AFB_1_-BSA and blocked as described above. AFB_1_ standard solutions with a range of concentrations of 0.05–50 ng/mL were added to each well. Then, 50 μL of soluble scFv was added to each well. The plates were incubated at 37 °C for 1 h and washed with PBST. Protein A/HRP was incubated at 37 °C for 1 h, aspirated, and then 100 μg/mL TMB was added, and the absorbance was recorded at 450 and 650 nm. The half-maximal inhibitory concentration (IC_50_) of free AFB_1_ was determined.

## 4. Discussion

Insufficient stability of the VH-VL interface of scFv has often been suggested as the main cause of irreversible scFv inaction, since transient opening of the interface exposed hydrophobic patches that favor intermolecular aggregation [1]. The VH domain of one chain was suggested to pair with the VL domain of another chain and *vice versa*. Besides the external factors, the interface stability affected protein aggregation behavior deeply, such as the linker between two domains [23,24] and the residues at the interface [3,5]. In this paper, we focused on scFv stabilization by an interdomain disulfide bond.

Molecular modeling revealed several possible disulfide bond sites at the interface of VH (H4) and VL (H4) and evaluated the Cα-Cα distance. Previous studies have shown that the Cα-Cα distance of disulfide bonds between cysteine residues in known proteins ranges from 4.2 Å to 6.6 Å [9,10]. We focused on FR H4 and FR L2 or FR H2 and FR L4 as the target segments for the introduction of interdomain disulfide bonds, which serve as the interface between the VH (H4) and VL (H4) domains. The effect of a disulfide bond on affinity should be taken into consideration, and molecular docking might be useful in predicting the changes in binding behavior.

The interdomain disulfide bonds enforce interface stability in the same molecule and prevent domain dissociation and subsequent intermolecular aggregation. The decreased extent of aggregation that we observed indicated that the interdomain disulfide bond between H44 and L100 improved interdomain stability. Multidomain proteins typically show complex equilibrium unfolding behaviors [3]. ScFv (H4) showed a single two-state unfolding transition, similar to a single-domain protein, and the stability of the whole protein was higher than that of any of the constituent domains alone, thereby indicating that the constituent domains of scFv are stabilized significantly by their interactions with other domains. The unfolding of recombinant antibodies can be described by a model in which the stability of each domain within the multidomain assembly is the sum of its intrinsic stability and the additional stabilization it experiences through its interactions with neighboring folded domains [25]. The interdomain disulfide bond largely increased additional stabilization, which has been proven in Fab [3].

The interdomain disulfide bond between H44 and L100 has a negligible effect on the binding between antibody and antigen. It was critical to choose an appropriate position to introduce the disulfide bond. According to the results of competitive ELISA, the disulfide bond did not affect affinity in our case. Molecular docking between scFv (H4) and AFB_1_ revealed that the binding sites of scFv (H4) were in the CDRs L3 and H3, while the interdomain disulfide bond was designed between FRs H2 and L4. The sufficient distance from the disulfide bond to the binding sites might be the reason for the negligible effect of the interdomain disulfide bond on affinity.

## 5. Conclusion

The scFv (H44-L100) were stabilized with an interdomain disulfide bond, and the stability of the scFv (H4) increased from a GdnHCl_50_ of 2.4 M to 4.2 M after addition of the H44-L100 disulfide bond. An affinity assay indicated that scFv (H4) and the scFv (H44-L100) mutant had similar IC_50_ values and affinity to AFB_1_.The stability of H4 increased after addition of the H44-L100 disulfide bond. Since the conformation of scFv is relatively conservative, especially the framework segments, our results confirm that the interdomain disulfide bonds could stabilize scFv without affecting affinity.

## Figures and Tables

**Table 1:** Cα-Cα distance between residues in the framework regions of VH and VL in the scFV(H4) model structure.

Site Pair	Locations	Distance (Å)
H44-L100	FR H2, FR L4	5.36
H46-L98	FR H2, FR L4	6.23
H101-L44	FR H4, FR L2	6.58
H103-L42	FR H4, FR L2	6.98
H103-L43	FR H4, FR L2	5.73

**Table 2:** Primers used in the scFv cloning and disulfide bond construction.

Primer	Sequence
LMB	CGACCCGCCACCGCCGCTG
pHEN	CTATGCGGCCCCATTCA
L100Back	ATGTGCGGCCGCCCGTTTGATTTCCACCTTGGTCCCACAGCC
H44For	GCTCCAGGGAAGTGTCTGGAGTGGGTC
H44Back	GACCCACTCCAGACACTTCCCTGGAGC
